# Actin-like Protein 6A Expression Correlates with Cancer Stem Cell-like Features and Poor Prognosis in Ovarian Cancer

**DOI:** 10.3390/ijms24032016

**Published:** 2023-01-19

**Authors:** Po-Ming Chen, Chui-Nguk Wong, Chui-Na Wong, Pei-Yi Chu

**Affiliations:** 1 Research Assistant Center, Show Chwan Memorial Hospital, Changhua 500, Taiwan; 2 Department of Obstetrics and Gynecology, Show Chwan Memorial Hospital, Changhua 500, Taiwan; 3 Department of Post-Baccalaureate Medicine, College of Medicine, National Chung Hsing University, Taichung 402, Taiwan; 4 Department of Pathology, Show Chwan Memorial Hospital, Changhua 500, Taiwan; 5 School of Medicine, College of Medicine, Fu Jen Catholic University, New Taipei 242, Taiwan; 6 National Institute of Cancer Research, National Health Research Institute, Tainan 704, Taiwan

**Keywords:** ovarian cancer, metastasis, ACTL6A, cell cycle, prognosis, resistance

## Abstract

Ovarian cancer has the highest mortality rate among gynecological cancers, often diagnosed at the late stage and lacking an effective targeted therapy. Although the study of malignant features of cancer, considered to be cancer stem cells (CSCs), is emerging, the aim of this study was to predict and explore the possible mechanism and clinical value of genetic markers in the development of ovarian cancer from a combined database with CSCs features. The common differentially expressed genes (DEGs) were selected in GSE185833 and GSE176246 datasets from the Gene Expression Omnibus (GEO). The GSE185833 dataset was created to reveal gene expression profiles of peritoneal metastasis tissues using single-cell sequencing, and the GSE176246 dataset was determined from gene expression profiles of chemotherapy-refractory ovarian cancer cell lines compared with ovarian cancer cell lines by RNA-seq analysis. By analyzing the correlation between common DEGs and prognosis of ovarian cancer and its possible pathways and functions were predicted by The Cancer Genome Atlas (TCGA) database. The expression levels of 11 genetic markers were significantly elevated in highly invasive and chemoresistant ovarian cancer. The expression of Actin-like protein 6A (ACTL6A) was found to be correlated with survival prognosis, and the total survival time of the patients with high expression of ACTL6A was shorter than those with low expression. Gene set enrichment analysis (GSEA) showed that ACTL6A positively enriched the gene set of ‘Cell cycle’ and ACTL6A negatively enriched the gene set of focal adhesion. CP724714, a human epidermal growth factor receptor 2 (HER2) inhibitor, could serve as a therapeutic option when ACTL6A levels are high in ovarian cancer cells. The high expression of ACTL6A is a poor prognostic factor in ovarian cancer and may serve as an effective biomarker for predicting treatment-refractory, metastasis, and prognosis of patients with ovarian cancer. The use of HER2 inhibitors is a promising therapeutic strategy against chemoresistant ovarian cancer.

## 1. Introduction

Ovarian cancer has the highest mortality rate among gynecological malignancies since about 80% of ovarian cancer patients are diagnosed with late-stage disease and with a five-year survival rate of about 29% [1]. The standard treatment for advanced ovarian cancer undertakes cytoreductive surgery followed by the platinum-based chemotherapy (cisplatin or carboplatin) [2]. Although platinum-based chemotherapy is incredibly advantageous; however, many platinum-resistant tumors have proven unresponsive, especially metastatic recurrence, which inevitably causes most mortalities [3]. Herein, better understanding of the basis for advanced ovarian cancer, as well as identification of helpful biomarkers and development of diagnostic tools, useful intervention strategies or therapeutics are imperatively needed for this intractably disease.

Cancer stem cells (CSCs) are known to play a critical role in the regulation of epithelial-mesenchymal transition (EMT), metastasis, and self-renewal of CSCs, which generate new cancer cells by modulating various signaling pathways [4]. The various initializations of ovarian cancer progression are made more complex by tumor heterogeneity, which can be categorized into either inter-tumor or intra-tumor heterogeneity [5]. Although several surface markers used to identify ovarian CSCs, such as CD24 [6], CD44 [7], CD117 [8], and CD133 [9] can be targeted by many strategies, they do not satisfy the patients’ needs for treatments. 

The Gene Expression Omnibus (GEO) database has comprehensive archives of gene expression in the National Center of Biotechnology Information (NCBI) that provides a well-annotated database of freely distributed microarrays, next-generation sequencing, and other forms of high-throughput functional genomics data submitted by the research community [10]. The methods of mining the GEO database mainly include the screening of differentially expressed genes (DEGs) involved in various experimental designs [10]. Additionally, The Cancer Genome Atlas (TCGA) is a landmark cancer genomics program of the most large-scale sequencing database, which offers many cancer genomic datasets [10]. GEPIA (Gene Expression Profiling Interactive Analysis) is a web-based tool that delivers fast and customizable functionalities based on TCGA, which provides differential expression analysis, profiling plotting, correlation analysis, and patient survival analysis with an interactive web interface [11].

Actin-Like Protein 6A (ACTL6A, BAF53a) is a SWItch/Sucrose Non-Fermentable (SWI/SNF) regulatory complex protein with ATP dependent chromatin-remodeling factors [12]. ACTL6A is necessary and sufficient for neural progenitor and hemopoietic stem cell proliferation [13,14]. ACTL6A (BAF53a) is replaced by the homologous BAF45b, BAF45c, and BAF53b is essential for the transition from stem/progenitors to postmitotic cells [13]. However, the role of ACTL6A and ACTL6A-related mechanisms in advanced ovarian cancer remains poorly understood.

Therefore, we first combined GEO databases (GSE185833 and GSE176246) in the present study to mine the genetic markers involved in chemoresistance and metastasis, the possible mechanisms in the development of ovarian cancer using GSEA (http://www.linkedomics.org/login.php, data obtained on 1 October 2022) and to further identify the genetic molecular markers associated with the prognosis by Kaplan-Meier Plotter (https://kmplot.com/analysis/index.php?p=service&cancer=ovar, data obtained on 1 October 2022). In this study, the expression of ACTL6A gene in chemoresistant and metastatic ovarian cancer was mined, we and further performed a drug screening based on ACTL6A expression in different ovarian cancer cells, which provided the potential association of high ACTL6A expression levels with the good response of CP724714, a human epidermal growth factor receptor 2 (HER2) inhibitor, discovered through Q-omics software analysis.

## 2. Results

### 2.1. GEO Datasets Filter the Common DEGs

**Figure 1:**
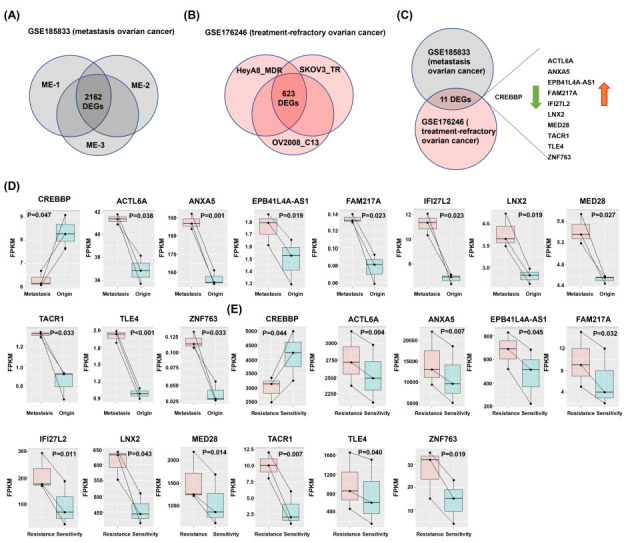
The common genes associated chemoresistance and metastasis in ovarian cancer. (**A**) The Venn diagram shows the mapping results among ovarian 2162 common genes associated metastasis in the GSE185833 dataset. (**B**) The Venn diagram shows the mapping results among ovarian 623 common genes associated chemoresistance in GSE176246 dataset. (**C**) The Venn diagram shows the mapping results among ovarian 11 common genes associated chemoresistance and metastasis in GSE185833 and GSE176246 datasets. (**D**) The mRNA expression levels of 11 DEGs, including CREBBP, ACTL6A, ANXA5, EPB41L4A-AS1, FAM217A, IFI27L2, LNX2, MED28, TACR1, TLE4, and ZNF763 between the peritoneal metastasis tissues and their corresponding primary ovarian cancer. (**E**) The mRNA expression levels of 11 DEGs, including CREBBP, ACTL6A, ANXA5, EPB41L4A-AS1, FAM217A, IFI27L2, LNX2, MED28, TACR1, TLE4, and ZNF763 between the chemoresistant ovarian cancer cells and their corresponding parental ovarian cancer cells.

A total of 2162 DEGs were found between peritoneal metastasis tissues and ovarian tumor tissues from GSE185833 using a Venn diagram (Figure 1A). As shown in Figure 1B, 623 genes were significantly differentially expressed between the chemoresistance cell lines and the non-chemoresistance cell lines in GSE176246 using a Venn diagram (Figure 1B). The 11 common DEGs were mined in GSE185833 and GSE176246 datasets (Figure 1C). Then the repertoires of 11 DEGs mRNA expression were simultaneously visualized between metastasis and origin tumor by paired t-test method. The results showed that there were 10 up-regulated DEGs between metastatic and primary tumors, including ACTL6A, ANXA5, EPB41L4A-AS1, FAM217A, IFI27L2, LNX2, MED28, TACR1, TLE4, and ZNF763 (Figure 1C), and conversely, CREBBP was down-regulated between metastatic and primary tumors (Figure 1C), as well as in the resistant cancer cells (Figure 1D). The 11 common DEGs, with 10 up-regulated and one down-regulated genes, could have the effects of chemotherapy and metastasis on GSE185833 and GSE176246 datasets.

### 2.2. ACTL6A Expression Associates with Poor Prognosis and Up-Regulates in Ovarian Sphere Cancer Cell

**Figure 2:**
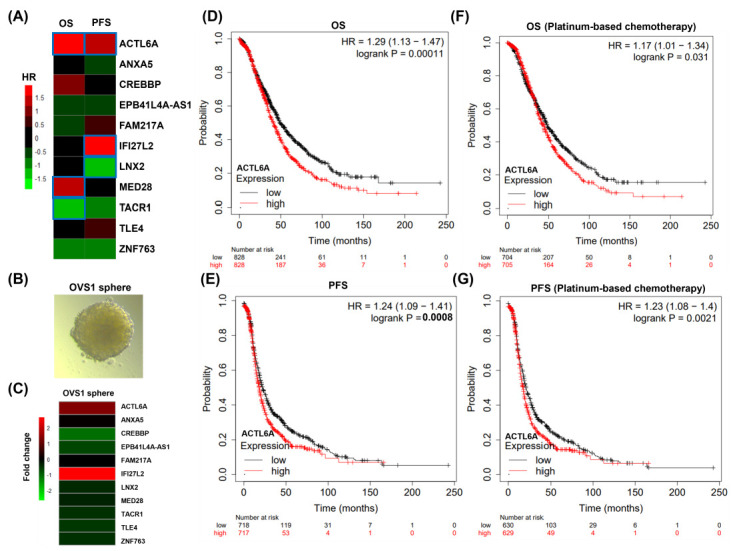
GACTL6A has poor prognosis for overall survival (OS) and progression-free survival (PFS) (**A**) Heat map of hazard ratios (HR) illustrating cancer-11 DEGs with altered prognosis. We selected the median as the suitable expression threshold for splitting the high-expression and low-expression cohorts. The hazards ratio (HR) was calculated based on the Kaplan–Meier survival method, and the HR value was scaled in decibel. (**B**) Formation of spheres under the stem cell selective condition on day 8 after culturing from parental OVS1. (**C**) Changes in gene expression of CREBBP, ACTL6A, ANXA5, EPB41L4A-AS1, FAM217A, IFI27L2, LNX2, MED28, TACR1, TLE4, and ZNF763 in a heat map. (**D**) All ovarian cancer patients revealed that patients with high expression of ACTL6A had worse overall survival (OS) compared to the ACTL6A low expression patients. (**E**) All ovarian cancer patients revealed that patients with high expression of ACTL6A had worse progression-free survival (PFS) compared to the ACTL6A low expression patients. (**F**) Chemotherapy ovarian cancer patients revealed that patients with high expression of ACTL6A had worse overall survival (OS) compared to the ACTL6A low expression patients. (**G**) Chemotherapy ovarian cancer patients revealed that patients with high expression of ACTL6A had worse progression-free survival (PFS) compared to the ACTL6A low expression patients.

Survival maps (Figure 2A), i.e., heat maps of hazard ratio (HR), showed the prognostic value of overall survival (OS) and progression-free survival (PFS) in ovarian cancer. We selected the medium value as the cutoff for splitting the high expression and low expression groups. The HR was calculated based on the Kaplan–Meier survival analysis, and the HR value was scaled in decibels. Statistically significant (*p* < 0.05) genes were framed with blue color (Figure 2A). As our knowledge, drug resistance and metastasis in malignant ovarian cancer for the enrichment of CSCs, thus we used OVS1, a cell line from Taiwanese ovarian cancer in stem cell selective conditions conducive to sphere formation (Figure 2B). Relative to their corresponding parental cells, 2 and 2 of the 11 DE genes were down-regulated and up-regulated in the sphere cells, respectively (Figure 2C), which were consistent with the two GEO datasets, apart from EPB41L4A-AS1. Considering both OS and PFS, the survival analysis demonstrated that high expression levels of ACTL6A was associated with a poor prognosis of ovarian cancer patients (*p* < 0.001 for OS and PFS, Figure 2D,E), as well as in ovarian cancer patients received chemotherapy (*p* = 0.031 for OS and *p* = 0.0021 for PFS, Figure 2F,G). 

### 2.3. ACTL6A Expression Has a Correlation with Cell Cycle Progression in Ovarian Cancer 

**Figure 3:**
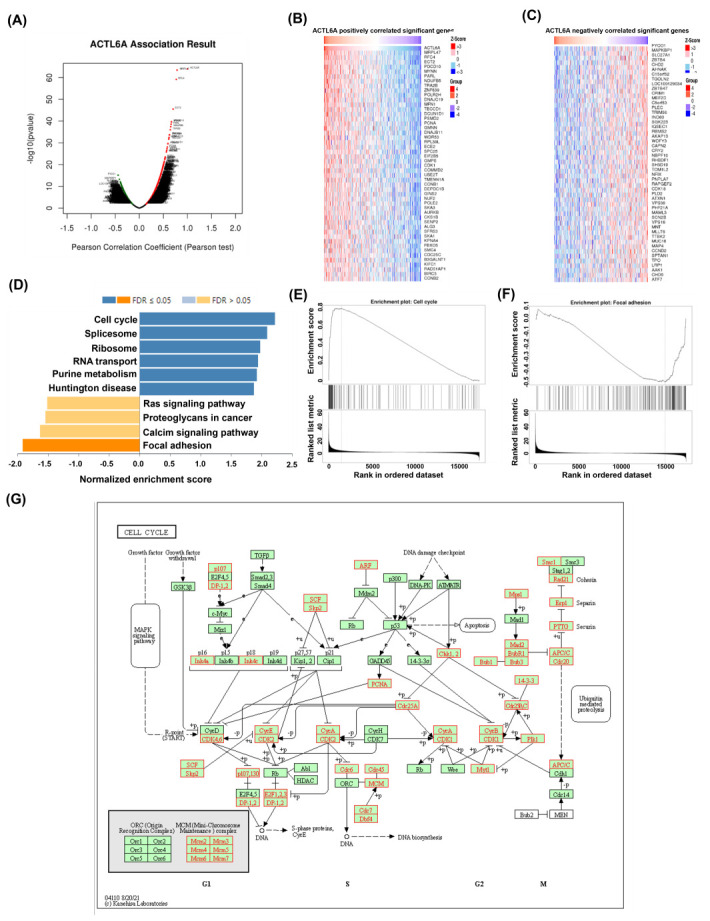
Most cell cycle genes in positive correlation and focal adhesion genes in negative correlation with ACTL6A in ovarian cancer. (**A**) Association results of ACTL6A in ovarian cancer (Number of patients = 303); Pearson test was performed to analyze LinkFinder. Volcano plots show the statistical association results of ACTL6A for all ovarian cancer DEGs. (**B**) Data for the top 50 positive association genes are visualized in a heat map. (**C**) Data for the top 50 negative association genes are visualized in a heat map. (**D**) In the bar plots, the color scale represents the FDR value, and the length of the bar represents the normalized enrichment score (NES) value: positive correlated, blue Number of patients = 303 > 0; and negative correlated, orange < 0. (**E**) GSEA results showing cell cycle is differentially enriched pathway in ACTL6A-related genes. NES, normalized enrichment score. (**F**) GSEA results showing focal adhesion is differentially enriched pathway in ACTL6A-related genes. NES, normalized enrichment score. (**G**) KEGG pathway annotations of the cell cycle pathway.

The LinkFinder module of LinkedOmics was showed the association genes of ACTL6A based on the data of 303 ovarian cancer patients. The platform was selected as HiSeq RNA in both the Search dataset and the Target dataset (ID-17198). A volcano plot showed that the results of the correlation analysis should consider both the significance and the *p*-value of the Pearson correlation coefficient were obtained from the statistical method, and had more positive correlation genes than the negative genes with ACTL6A. (Figure 3A), The top-rank significant genes positively associated with ACTL6A was shown in the heat map (Figure 3B), and the top-rank significant genes negatively associated with ACTL6A was shown in the heat map (Figure 3C). KEGG (http://www.genome.jp/, data obtained on 1 October 2022) is a knowledge database for the mapping of specific pathways to the large of DEGs. The bar plot showed a summary of significant enriched KEGG pathway indicated that genes differentially expressed in positive correlation with ACTL6A was involved mainly in cell cycle, spliceosome, ribosome, RNA transport, purine metabolism, and Huntington disease, and negative correlation with ACTL6A was involved mainly in focal adhesion (Figure 3D). The cell cycle and focal adhesion pathways were also selected as the interesting terms and the GSEA results are shown in detail in Figure 3E,F. Referring to cell cycle signaling pathway, we determined the positive relationship between ACTL6A and the specific genes with red color (Figure 3G). 

### 2.4. CP734714, a HER2 Inhibitor as a Therapeutic Option in the Context of High ACTL6A

**Figure 4:**
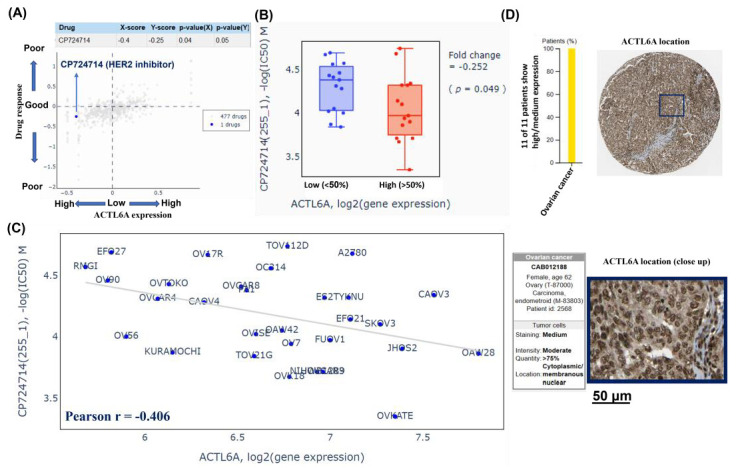
CP724714 as a therapeutic option in the context of high ACTL6A in ovarian cancer. Q-omics analysis was used to analyze cross-association scores for the identification of candidate drugs acting on ovarian cancer cells based on ACTL6A expression. (**A**) X-score: log(fold-change) of ACTL6A expression between samples of high and low response of target drug. Y-score: log(fold-change) of target drug response between samples of high and low ACTL6A expression. The blue dot hits a negative association with *p*-value(X) < 0.05 and *p*-value(Y) < 0.05. (**B**) The boxplots of −log (half maximal inhibitory concentration (IC50)) M of CP724714 in low (<50%) and high (>50%) ACTL6A expression. Fold-change: the difference of IC50 variable between low (<50%) and high (>50%) ACTL6A expression (**C**) The scatter plot showed that ACTL6A expression levels and IC50 of CP724714 in 30 ovary adenocarcinoma cell lines. The r value was calculated by Pearson’s correlation. (**D**) The yellow bar showed that 11 of 11 ovarian cancer patients show high/medium expression. The representative immunohistochemistry (IHC) staining of ACTL6A molecule with specific ACTL6A (CAB012188) antibody in a patient with ovarian cancer. Scale bar = 50 μm.

To explore potential pharmaceutical approaches that can effectively target ovarian cancer with high ACTL6A expression, we used the Q-omics analysis (http://qomics.sookmyung.ac.kr/, data obtained on 1 October 2022) to search potent drugs that may act to treat with high ACTL6A expression. We operated cross-associations between drug response and ACTL6A expression in 30 ovary adenocarcinoma cell lines. The results showed in Figure 4A that one out of 478 drugs has the statistical significance between low and high ACTL6A expression groups. The 15 ovarian cancer cells with high ACTL6A expression had low log (IC50) values in an optimal response to CP734714 (Figure 4B). The IC50 of CP734714 has a negative correlation with ACTL6A expression (Pearson r = −0.406), supporting the notion that CP734714 serves as a therapeutic option in the context of high ACTL6A expression. The human protein atlas (HPA) analysis was used to observe that ACTL6A protein expressions in ovarian cancer (https://www.proteinatlas.org/ENSG00000136518-ACTL6A/pathology/ovarian+cancer#Quantity, data obtained on 1 December 2022). The results showed that 11 of 11 malignant ovarian tumors displayed moderate to strong nuclear, cytoplasmic, and membrane immunoreactivity positivity of ACTL6A protein (Figure 4D).

## 3. Discussion

We identified 11 DEGs between metastasis and chemo-refractory of ovarian cancer through searching two different data series (GSE185833 and GSE176246) relevant to ovarian cancer. Of the 11 identified DEGs, ACTL6A expression was found to play important roles in ovarian cancer progression and to be closely correlated with adverse prognosis for overall survival (OS) and progression-free survival (PFS) in ovarian cancer patients.

Although other 10 identified DEGs expressions were not correlated with prognosis, including CREBBP, ANXA5, EPB41L4A-AS1, FAM217A, IFI27L2, LNX2, MED28, TACR1, TLE4, and ZNF763, they may act as cancer stem cells (CSCs) markers, especially CREBBP and IFI27L2 (Figure 2C). A previous study reported that earlier loss of CREBBP is advantageous for malignant stem cell properties on lymphoid progenitors [15].

Our study found the relationship of ACTL6A and the advanced ovarian cancer cells in chemoresistance, metastasis, poor prognosis, and CSCs. The uncovered mechanisms revealed that ACTL6A mainly had positive enrichment of cell cycle and negative enrichment of focal adhesion. Unlike most of the published reports showing that several surface markers used to identify ovarian CSCs, such as CD24 [6], CD44 [7], CD117 [8], and CD133 [9], we have unveiled the ACTL6A had poor prognosis and correlated enrichment of CSCs in ovarian cancer.

Previous studies showed that overexpression of ACTL6A maintains an aggressive mesothelioma cancer cell phenotype by reducing p21 expression [16] and promotes the proliferation in NSCLC by regulating Hippo/YAP pathway [17]. Additionally, ACTL6A increases colon cancer invasion, metastasis, and epithelial mesenchymal transition (EMT) [18] and it was suggested that ACTL6A could be a regulator of enforces the progenitor state and a potential therapeutic target in hepatocellular carcinoma (HCC) [19,20,21]. Previous research demonstrated that follicle-stimulating hormone (FSH)-enhanced glycolysis in ovarian cancer contained a high level of ACTL6A and that ACTL6A increased phosphoglycerate kinase 1 (PGK1), therefore aiding the proliferation of ovarian cancer [22]. Elevated expression of ACTL6A is associated with cancer stem cell-like features, advanced stages of ovarian tumors and poor survival of patients.

We have taken the list of ACTL6A-correlated genes associated with ovarian cancer pathway from KEGG database. These genes are associated with ‘cell cycle’, ‘spliceosome’, ‘RNA transport’, ‘purine metabolism’, and ‘Huntington disease’. As some spliceosome gene mutations can be translated immune-related precursor messenger RNA (pre-mRNA) into multiple protein products that can have unique functions result in immune dysregulation and cancer development [23]. Additionally, many studies elucidating the relationships between malignant transformation and metastasis and cellular adhesion processes, and cell-matrix adhesions have important biological processes including cell motility, cell proliferation, cell differentiation, regulation of gene expression and cell survival [24]. In this study, we proposed that the common gene, ACTL6A, was selected from the datasets of features of the ovarian cancer stem-like cell made by Venn diagram approaches, and it was positively correlated with cell cycle progression. Conversely, the malignant epithelial cell migration started through attenuation of cell adhesion and focal adhesion formation [25].

CP724714, a selective (ErbB2; Her-2/neu) tyrosine kinase inhibitor to inhibit ErbB2 receptor autophosphorylation in intact cells, has been used in breast cancer cell lines [26] and SKOV3 ovarian cancer cells [27], which had significant decline on both survival after treated with CP724714. However, studies into CP724714 prescription adhering to the principles of precision treatment are lacking. It is noteworthy that the presentation reveals CP724714 as a potential therapeutic treatment for ovarian cancer in the presence of high ACTL6A expression levels. More evidence needs to be validated, this result could support the development of the precision medicine for ovarian cancer based on high ACTL6A expression levels.

Overall, the present study indicated that the expression of marker of ACTL6A as a poor prognostic factor predicted metastasis and prognosis of ovarian cancer patients. ACTL6A expression elevated within the chemoresistance and metastasis of ovarian cancer and cancer stem-like cells. Clinical trials of HER2 inhibitors are needed to confirm the results of the present study.

## 4. Materials and Methods

### 4.1. Public Database Analyses

We selected GSE185833 and GSE176246 datasets from the GEO database. The GSE185833 dataset consists of the ovarian cancer patients. Each pair of samples represents a single patient’s the peritoneal metastasis tissue and primary ovarian cancer tissue (OC-1 and ME-1; OC-2 and ME-2; OC-3 and ME-3) [28]. The GSE176246 dataset consisted of isogenic pairs of ovarian cancer cell lines OV2008 (chemosensitive) and C13 (cisplatin resistant) cells derived from OV2008; HEYA8 (chemosensitive) and HEYA8 MDR (carboplatin-resistant) and isogenic taxol-sensitive SKOV3 and taxol-resistant SKOV3 TR cells were used for the study [29]. Then, we used the paired *t*-test for *p*-value as the threshold to find the common differential genes.

### 4.2. Web Server Survival Analysis

The survival analysis, 11 DEGs (as shown in Figure 1C) mRNA expression in this study, was performed using the web server for the Kaplan–Meier plots from RNA gene chip datasets by selecting the median values between the lower and upper quartiles into high group and low group. Please have a look at (https://kmplot.com/analysis/index.php?p=service&cancer=ovar, data obtained on 1 October 2022).

### 4.3. LinkedOmics Database Analysis

The LinkedOmics database (https://www.linkedomics, data obtained on 1 October 2022) is mainly used to analyze TCGA ovarian cancer dataset (ID-127570) using a comprehensive online platform [30]. We analyzed the co-expression of ACTL6A on the heat map through the LinkedOmics function module using Pearson’s correlation coefficient. We further analyzed ACTL6A-related enrichment through gene set enrichment analysis (GSEA) which were depended on the Molecular Signatures Database (MSigDB). The rank norm was FDR < 0.05, and 500 simulations were performed.

### 4.4. Cell Culture and Sphere Forming Assay

Monolayer cells of parental OVS1 cells were cultured in the stem cell selective condition by plating cells in Corning Costar ultra-low attachment 6-well plates (Sigma-Aldrich Inc., St Louis, MO, USA) at a density of 1 × 10^5^ cells per well with 3 mL of serum-free PSGro hESC/iPSC growth medium (System Biosciences, Palo Alto, CA, USA). Propagation of spheres was processed by gentle centrifugation, dissociation with trypsin-EDTA and repeated pipetting to obtain single-cell suspensions every 5–8 days, and then plating the cells in the above stem cell selective condition. The spheres were found under microscope after 10 days of culturing. Detailed procedures of total RNA isolation and RNA sequencing were described elsewhere [31].

### 4.5. Q-Omics Drug Database

The drug sensitivity profiling based on ACTL6A expression was analyzed using the microarray data in Q-omics v.1.01 software (accessed on 1 October 2022) (http://qomics.sookmyung.ac.kr/) [32]. The cell line analyses available to Q-omics are as follows: (1) Cross-association analyses between any pair of datasets according to ACTL6A expression and drug screening data; (2) Box plot/scatter analyses of pairs according to ACTL6A expression and IC50 of CP734714.

### 4.6. Human Protein Atlas

The differential expression levels of ACTL6A protein in ovarian cancer tissues was validated using the Human Protein Atlas (HPA) portal. Immunohistochemistry showed that ACTL6A protein localization and expression in cytoplasm, membrane, and nuclei of ovarian cancer tissues by CAB012188, a specific ACTL6A antibody, and the results is publicly available at https://www.proteinatlas.org/ENSG00000136518-ACTL6A/pathology, data obtained on 1 December 2022).

### 4.7. Statistical Analysis

Differences of CREBBP, ACTL6A, ANXA5, EPB41L4A-AS1, FAM217A, IFI27L2, LNX2, MED28, TACR1, TLE4, and ZNF763 expression of each patient’s peritoneal metastasis tissue and primary ovarian cancer tissue (OC-1 and ME-1; OC-2 and ME-2; OC-3 and ME-3) and isogenic pairs of ovarian cancer cell lines (OV2008 and C13; HEYA8 and HEYA8 MDR; SKOV3 and SKOV3 TR) were analyzed with the paired *t*-test using SPSS software (version 13.0; SPSS, Inc., Chicago, IL, USA). Overall survival (OS) and progression-free survival (PFS) rates were estimated using the Kaplan-Meier method. A box plot was used to visualize differences in the IC50 of CP734714 between low and high ACTL6A groups. The differences between two groups were analyzed by calculating the fold-change using Student’s *t*-test. *p* < 0.05 was considered statistically significant.

## 5. Conclusions

The present study demonstrated a novel strategy that used the common DEGs from the datasets of ovarian cancer metastasis and drug-refractory. Furthermore, it is noteworthy that ACTL6A expression was up-regulated in ovarian cancer sphere cells. ACTL6A was considered to be a novel prognostic marker for ovarian cancer. CP724714 was in the modulation of ACTL6A may serve as a potential novel therapeutic strategy for ovarian cancer.

In other words, targeting the ACTL6A-related pathway, especially with ‘cell cycle’ inhibition, suggested the use of HER2 inhibitors to be promising in ovarian cancer.

## Data Availability

The data presented in this study are available on request from the corresponding author.
